# A Straightforward Approach for Synthesizing Electromechanical Sigma-Delta MEMS Accelerometers

**DOI:** 10.3390/s20010091

**Published:** 2019-12-22

**Authors:** Dongliang Chen, Liang Yin, Qiang Fu, Wenbo Zhang, Yihang Wang, Guorui Zhang, Yufeng Zhang, Xiaowei Liu

**Affiliations:** 1 MEMS Center, Harbin Institute of Technology, Harbin 150001, China; zoom_chen@126.com (D.C.); 16B321002@hit.edu.cn (W.Z.); grzhit@126.com (G.Z.);; 2 Key Laboratory of Micro-Systems and Micro-Structures Manufacturing, Harbin Institute of Technology, Harbin 150001, China; 3 State Key Laboratory of Urban Water Resource & Environment, Harbin Institute of Technology, Harbin 150001, China

**Keywords:** accelerometer, MEMS, sensor interface, electromechanical sigma-delta

## Abstract

The EM-ΣΔ (electromechanical sigma-delta) approach is a concise and efficient way to realize the digital interface for micro-electromechanical systems (MEMS) accelerometers. However, including a fixed MEMS element makes the synthesizing of the EM-ΣΔ loop an intricate problem. The loop parameters of EM-ΣΔ can not be directly mapped from existing electrical ΣΔ modulator, and the synthesizing problem relies an experience-dependent trail-and-error procedure. In this paper, we provide a new point of view to consider the EM-ΣΔ loop. The EM-ΣΔ loop is analyzed in detail from aspects of the signal loop, displacement modulation path and digital quantization loop. By taking a separate consideration of the signal loop and quantization noise loop, the design strategy is made clear and straightforward. On this basis, a discrete-time PID (proportional integral differential) loop compensator is introduced which enhances the in-band loop gain and suppresses the displacement modulation path, and hence, achieves better performance in system linearity and stability. A fifth-order EM-ΣΔ accelerometer system was designed and fabricated using 0.35 μm CMOS-BCD technology. Based on proposed architecture and synthesizing procedure, the design effort was saved, and the in-band performance, linearity and stability were improved. A noise floor of 1 $\mu g/\sqrt{\mathrm{Hz}}$, with a bandwidth 1 kHz and a dynamic range of 140 dB was achieved.

## 1. Introduction

In the most recent decade, the readout interface circuit for micro-electromechanical systems (MEMS) sensors has been evolving toward digitalization [1,2,3]. It is simple to realize the digitalization by cascading an A/D converter. However, this discrete implementation is power and area consuming. Moreover, the processing and transferring of a weak analog signal is intricate and susceptible to outside interference [4,5].

The EM-ΣΔ (electromechanical sigma-delta) approach which incorporates the MEMS sensing element into a ΣΔ modulating loop is the most promising approach to realize the digitalization of sensor interface. The attractiveness comes from the fact that it is a concise implementation and fulfills the digitalization at the very front-end of the whole system; thus, it gives more latitude for digital processing. Moreover, the use of one-bit feedback linearizes the inherent second-order relationship between voltage and electrostatic force effectively [6,7,8,9,10].

Despite the advantages mentioned above, there are still obstacles for a simple implementation. The obstacles mainly come from the MEMS sensing element. Since the limited in-band gain is provided by the MEMS sensing element [11], the total in-band characteristic is degenerated compared to a purely electrical ΣΔ modulator [8]; thus, high-order modulation is inevitable. Meanwhile, in order to suppress the Brownian noise, the sensing element is typically packaged in a vacuum cavity [9,11], resulting a highly under-damped subsystem. Moreover, this second order section is fixed and the inside first-order node (which is velocity) is inaccessible for an electrical circuit [12]. These negative factors mixed together seriously aggravate the synthesizing of loop parameters, which is already a complicated problem for a purely electrical ΣΔ modulator.

This problem can be divided into two aspects. First is the stabilization of the EM-ΣΔ loop. For an electrical ΣΔ loop, the proof of stability has not been rigorously established; approximate models and empirical observations are used for stability check [8,13]. The situation is worse for EM-ΣΔ, since there is an unchangeable highly under-damped MEMS structure. For most researchers [7,8,9,14] the first thing is to degenerate this second order section into a first-order one by introducing an adjustable zero. Then, the traditional design strategy for electrical ΣΔ can be used. However, this approach will introduce a pole located at the origin as well, which will severely impair the low frequency loop gain [8,12]. Thus, there is an inevitable trade-off between the effectiveness of the phase compensator and loop performance.

The second aspect is synthesizing the desired noise transfer function (NTF) derived from traditional methodology [15]. However due to the unchangeable nature of the MEMS transfer function, there is one degree-of-freedom(DOF) lost in the synthesizing procedure; thus, not all NTFs can be successfully synthesized [12,16]. The traditional approach is relied on for empirical experiments; the synthesis is a “trail-and-error” procedure which costs a lot of simulation time [17,18]. To solve this problem some researchers [12,16,19] proposed an unconstrained topology, where an extra feed-forward path is introduced to provide an extra DOF. However this approach is limited to a certain topology and is not widely adopted. Besides, some researchers [13,17,18] resort to an automatic way to facilitate the “trail-and-error” procedure. In these approaches, a genetic algorithm is used for searching the optimal solution to the synthesizing problem [13,17,18]. However the convergence and search direction is a problem; thus, manually intervention is inevitable. Moreover, the intuitions on the system behavior and critical trade-offs can not be established clearly that way, which are important to the system designer.

In this paper, we propose a straightforward way to synthesize the EM-ΣΔ loop parameters. Unlike previous methods which consider the loop as a whole and straightforwardly follow the form established by the purely electrical ΣΔ modulator, we take a separate consideration of the signal loop and quantization noise loop of the EM-ΣΔ system. The intricate EM-ΣΔ loop is analyzed step by step in aspects of the signal loop, the local positive feed-forward path (resulting from displacement modulation effect) and the digital quantization noise loop. A fifth-order EM-ΣΔ is established using the proposed synthesizing method. The straightforward design strategy makes the parameter optimization more effective, and simulation iteration can be reduced. By a clear understanding of loop behaviors, a more effective, discrete-time PID (proportional integral differential) compensator was created, which effectively improves the stability and linearity.

This paper is organized as follows: Section 2 describes the system architecture of the EM-ΣΔ accelerometer. Section 3 analyzes and discusses of the feedback loop. Section 4 gives implementation details of the system. Section 5 presents and discusses the experimental results of proposed system. And the paper ends with conclusion in Section 6.

## 2. The EM-ΣΔ System

**Figure 1:**
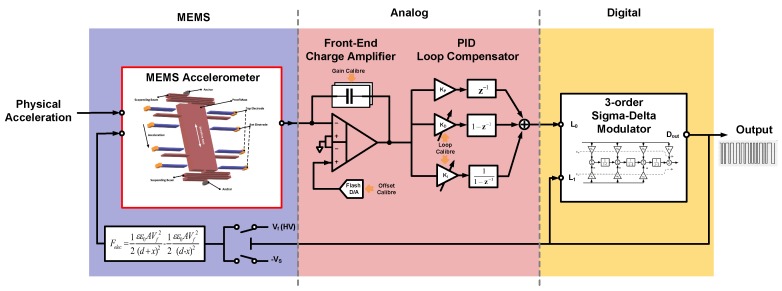
The architecture of the EM-ΣΔ system.

An EM-ΣΔ system is a hybrid system which contains elements across different domains. The architecture of the EM-ΣΔ system is shown in Figure 1.

As shown, the EM-ΣΔ accelerometer consists of three parts: (1) a MEMS sensing element transforms the physical input acceleration into electrical capacitance change ΔC; (2) an analog signal conditioning stage full-fills the amplification and loop compensation; (3) a 3-order electrical ΣΔ modulator realizes the conversion from analog to digital domain and provides the main noise shaping.

### 2.1. The MEMS Sensing Element

The MEMS sensing element is a critical part of the system, which constrains the applicable readout and feedback techniques, and affects the system’s performance and stability. A surface micro-machined capacitive MEMS accelerometer with a lateral comb-finger architecture is used as the front-end sensing element, due to its high sensitivity, low noise and the ease with which it establishes feedback loop through the electrostatic effect.

It exerts dominant influence on the readout circuit from two aspects:

(1) On the sensing direction: The sensing behavior of the MEMS element can be abstracted by a mass-spring-damping system, whose displacement change *x* can be expressed as:

(1) $$x=\frac{1}{s^{2}+\frac{\omega_{0}}{Q}s+\omega_{0}^{2}}\cdot a_{in},$$

where $\omega_{0}=\sqrt{k/m}$ and $Q=\sqrt{mk/b}$ are the resonating frequency and quality factor of the sensing element respectively, $a_{in}$ is the input acceleration, *m* is the mass of the movable proof mass, *b* is the damping factor introduced by air friction and *k* is the stiffness of the cantilever beam.

As the vacuum-packaging technique is typically used for reducing the Brawnian noise, the damping factor *b* is extremely low and the quality factor *Q* is pushed higher than 100 typically. Therefore, the stabilizing task is shifted to the following electrical part, which be come a knotty problem, especially for a high-order EM-ΣΔ.

(2) On the feedback direction: The feedback servo signal is exerted by the electrostatic effect of the parallel-plate sensing capacitor. However, the relationship of it is not linear, which can be expressed as:

(2) $$F_{elec}=\frac{C_{0}d_{0}V_{f}^{2}}{2{\left(d_{0}+x\right)}^{2}},$$

where $F_{elec}$ is the resultant electrostatic force due to the feedback voltage $V_{f}$. $C_{0}$ and $d_{0}$ are the initial sensing capacitance and displacement of the MEMS structure. *x* is the real-time displacement change due to external excitation.

It can be found from Equation (2) that, the displacement change *x* will modulate the electrostatic force $F_{elec}$. This unwanted side-effect (referred to as displacement modulation effect here) is important to the closed-loop operation. It not only degrades the closed-loop performance, but also introduces a local positive feed-forward path which will impair the system stability. The displacement modulation effect is discussed in next section in detail.

In a closed-loop configuration, the sensitivity of proposed EM-ΣΔ system is determined by the balance of input acceleration and electrostatic servo force, and it can be expressed as:

(3) $$ma_{in}=\frac{C_{0}V_{f}^{2}}{2d_{0}}\frac{V_{out}}{V_{S}}$$

(4) $$Sensitivity=\frac{V_{out}}{a_{in}}=\frac{C_{0}V_{f}^{2}}{2md_{0}V_{S}},$$

where $V_{out}$ is the system output voltage and $V_{S}$ is the quantization voltage level of the one-bit quantizer. Obviously, the closed-loop sensitivity is independent of damping and stiffness factors which are vulnerable to circumstance variation. And the second-order voltage-force relationship has been linearized by the use of one-bit feedback force.

### 2.2. The Analog Front-End

The analog front-end generally contains two sections: an amplification stage and a loop compensator.

The amplification stage provides voltage conversion with a proper gain. It is implemented by a SC (switch capacitor) charge amplifier, which detects the differential change of the MEMS sensing capacitor.

The displacement change *x* to the capacitance change ΔC can be expressed as:

(5) $$K_{x-C}=\frac{C_{0}d_{0}}{d_{0}-x}-\frac{C_{0}d_{0}}{d_{0}+x}\approx \frac{C_{0}x}{d_{0}}.$$

In the system bandwidth, the front-end detecting gain $K_{C-V}$ could be seen as a constant, which is given by:

(6) $$K_{C-V}=\frac{V_{S}}{C_{f}}K_{post},$$

where $V_{S}$ is the pre-charge voltage of the capacitance bridge, $C_{f}$ the feedback capacitance of front-end charge amplifier and $K_{post}$ is the total gain introduce by correlated double sampling and succeeding the amplifying stage.

The loop compensator, traditionally, is implemented by a phase-lead filter to introduce an additional phase lead to compensate for the excessive phase shift introduced by the sensing element [8,9,17,18]. However it would lead to a loss in the in-band loop gain and cause a series of problems. Thus we propose to use a discrete time PID (proportional integral differential) compensator. The design of loop filter is discussed in the following section.

### 2.3. The Electrical ΣΔ Loop Filter

The electrical ΣΔ loop filter realizes the digitalization of the analog signal and provides main part of quantization noise modulation. The selection of ΣΔ modulating order is a compromise between the sensing element and circuit performance, which stems from the following aspects [6,11,15]:

1. The sensing element, although providing a second order filtering characteristic, has a very limited in-band gain. It is not competent at suppressing the quantization noise alone, especially when high-voltage feedback is used.
2. The ΣΔ modulating loop faces a more serious stability problem as the order goes higher. More seriously, a vacuum packaged MEMS sensing element is used.
3. The low order ΣΔ exhibits more limit cycles, which will induce an early instability due to the displacement modulation effect.

**Figure 2:**
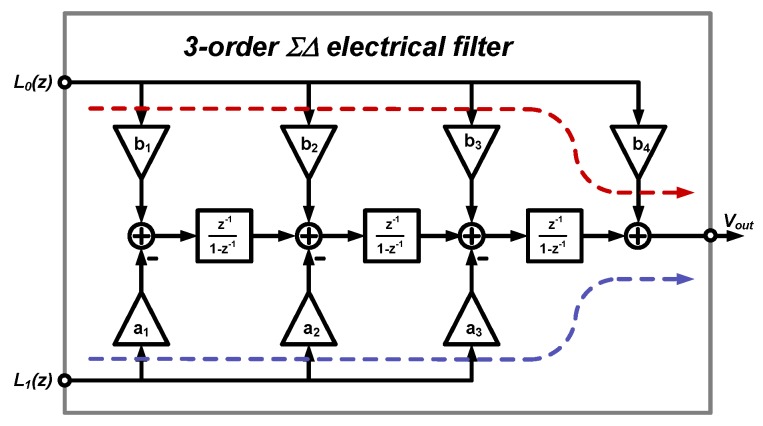
The architecture of the third-order electrical ΣΔ loop filter.

Thus, the order of the electrical ΣΔ loop filter used was chosen to be three. Together with the second-order sensing element, a fifth-order EM-ΣΔ is established. Its architecture is shown in Figure 2. The detailed deduction of its transfer function can be found in [20].

## 3. The Analysis of Control Loops

The synthesizing of an EM-ΣΔ system is a more difficult problem compared to an electrical ΣΔ modulator or a purely analog closed-loop. The difficulty comes from many aspects:

1. The sensing element is highly under-damped, which is easily self-excited.
2. The residue displacement introduces a local feed-forward path, which aggravates the stability problem.
3. The whole system is a high-order ΣΔ loop, whose first two stage cannot be tuned, and only an approximately linear model is available.

Previous designers [6,8,9,10,13,14,18] took the system as a whole ΣΔ modulator, following the traditional design routine of electrical ΣΔ loop. Thus, these problems mixed together, and a lot of time-consuming “trail-and-error” examinations were needed.

**Figure 3:**
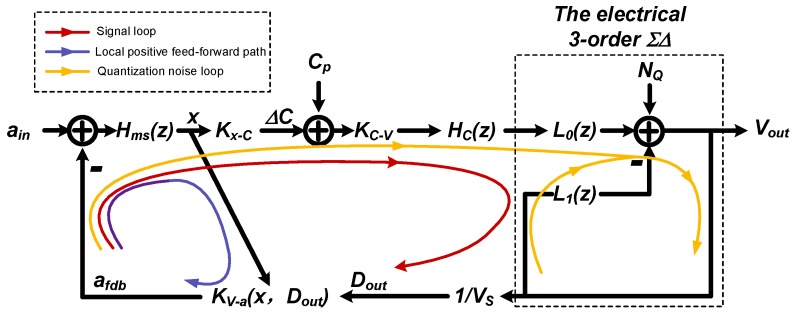
The abstracted signal flow diagram of the whole system.

In this section we will try to clarify the mixture of those problem, and further, propose a straightforward design procedure which is more efficiently. The abstracted mathematical signal flow diagram of the system is shown in Figure 3.

Note that, the loop characteristic is different at the viewpoint of signal and quantization noise respectively. Thus we proposed to take a separate consideration about the loop characteristic, as noted on Figure 3. In addition, the displacement modulation effect will introduce a localized positive feed-forward path, also noted in Figure 3. As will be discussed, it is specially impactful when there is a mismatch at front-end.

Here, we propose: (1) ensuring the stability of the signal loop; (2) that at the foundation, further adjustment should be made, taking the localization positive feed-forward path into consideration, (3) checking the digital quantization noise loop, using existing ΣΔ design method.

### 3.1. The Stability of the Signal Loop

**Figure 4:**
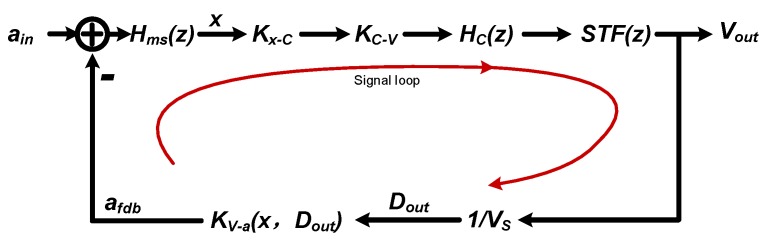
The signal flow diagram of the signal loop.

At the signal point of view, which is residue displacement here, the third-order electrical ΣΔ section is a “black-box” which could be expressed by its constant signal transfer factor $STF_{0}$. Thus, the signal flow diagram of the signal loop can be simplified as shown in Figure 4. The localized positive feed-forward path is not taken into consideration here, since it is a side-effect and focus should be put on the primary contradiction at this design stage.

The open-loop transfer function, which is an effective inspection of the stability, can be derived from Figure 4:

(7) $$LG\left(z\right)=\eta_{0}H_{ms}\left(z\right)H_{C}\left(z\right)K_{V-a}\left(x,D_{out}\right),\;\eta_{0}=\frac{K_{x-C}K_{C-V}STF_{0}}{V_{S}},$$

where $\eta_{0}$ is a loop constant which absorbs all constant terms. $H_{ms}\left(z\right)$ is the equivalent discrete-time transfer function of the mechanical sensing element, taking the timing effect into consideration, as described by X.S. Jiang [21]. $H_{C}\left(z\right)$ is the discrete-time transfer function of the loop compensator. $K_{V-a}\left(x,D_{out}\right)$ is the electrostatic feedback factor. When the residue displacement modulation is omitted here, $K_{V-a}\left(x,D_{out}\right)$ is degenerated to a constant $K_{V-a}$ From this point of view, it is obvious that the stabilization of the sensing element is mainly determined by the loop compensator $H_{C}\left(z\right)$.

As mentioned, previous researchers mainly resorted to a phase lead compensator, whose expression is shown in Equation (8). The transfer function $H_{C}\left(z\right)$ of traditional phase lead compensator is:

(8) $$H_{C}\left(z\right)=1-\alpha z^{-1},\;\alpha \in \left(0,1\right).$$

However, the in band gain provided by the compensator is 1−α. Obviously, the providing of high frequency phase lead is at the expense of significant losing of in-band gain. The situation is especially serious, when compensating a high-Q element, where an α higher than 0.9 is needed.

The insufficiency of in-band loop gain will cause problems in many aspects:

1. Control error is introduced, causing signal-dependent nonlinearity in the output.
2. The residue displacement can not be sufficiently suppressed, which will cause a stability problem through the localized positive feed-forward path.
3. The noise shaping ability of the mechanical path is reduced, resulting in an increased input referred quantization noise level.

In order to solve these problems, we introduce a new type of compensator for stabilizing the EM-ΣΔ signal loop, whose transfer function is in the form of:

(9) $$H_{C}\left(z\right)=K_{P}+K_{I}\left(\frac{1}{1-z^{-1}}\right)+K_{D}\left(1-z^{-1}\right).$$

**Figure 5:**
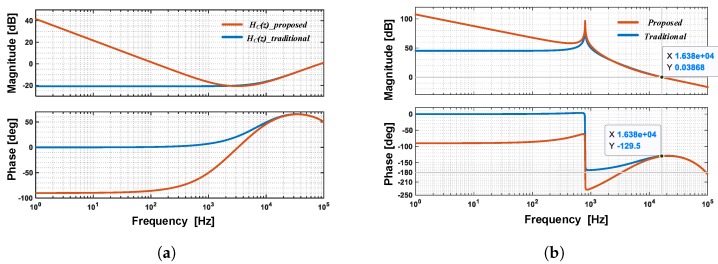
TA frequency response comparison with different type of compensator: (**a**) the frequency response of loop compensator $H_{C}\left(z\right)$; (**b**) the frequency response of total loop gain LG(z).

In the expression, there are three sections: a proportional section with a coefficient $K_{P}$, an integral section $1/(1-z^{-1})$ with a coefficient $K_{I}$ and a differential section $\left(1-z^{-1}\right)$ with a coefficient $K_{D}$. The proposed type of compensator presents a PID control characteristic, whose frequency response is shown in Figure 5a.

As shown in Figure 5a, at high frequency, the proposed compensator contributes the same phase lead compensation as the traditional one, which has a depth factor α=0.9. However, at low frequency, the in-band loop gain of proposed compensator is significantly larger—40 dB at 1 Hz, compared to the –20 dB decrement of a traditional compensator.

The frequency response of the total loop gain LG(z) with a different type of compensator is shown in Figure 5b. As shown, the proposed compensator achieves the same phase margin about $50^{\circ }$ as the traditional one, but in-band gain is greatly enhanced. It should be noted that there are multiple $-180^{\circ }$ crossings in the proposed loop. However, in the frequency range where the magnitude is larger than 0 dB, the number of positive crossings of $-180^{\circ }$ equals the negative crossings of $-180^{\circ }$. Thus the Nyquist stability criterion is satisfied, the signal loop is stable [22].

### 3.2. The Local Positive Feed-Forward Path

**Figure 6:**
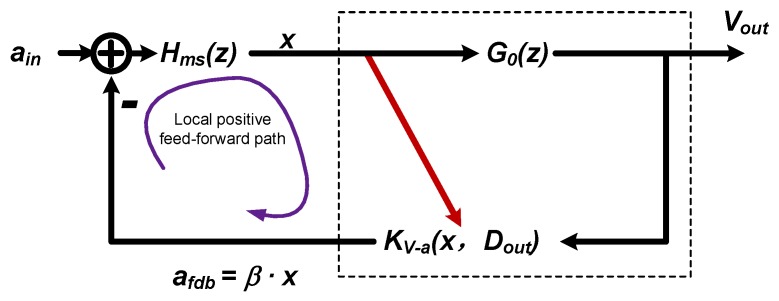
The simplified signal flow diagram to emphasize the local positive feed-forward path.

The local positive feed-forward path is caused by the displacement dependence of the electrostatic feedback force, referred to as displacement modulation effect. Thus, there is an implied feed-forward path from displacement *x* to the feedback factor $K_{V-a}$. The total feedback factor β is the summation of the signal path and the displacement modulation path. By rearranging Figure 3, the simplified signal flow diagram to emphasize the local positive feed-forward path is shown in Figure 6.

Where $G_{0}\left(z\right)$ is the lumped feed-forward gain of the electrical circuit:

(10) $$\begin{array}{c} G_{0}\left(z\right)=\eta_{0}H_{C}\left(z\right)=\frac{C_{0}K_{post}STF_{0}}{d_{0}C_{f}}\left[K_{P}+K_{I}\frac{1}{1-z^{-1}}+K_{D}\left(1-z^{-1}\right)\right] \end{array}.$$

And $K_{V-a}$ is the electrostatic factor with displacement modulation taken into consideration, which can be written as:

(11) $$\begin{array}{cc} K_{V-a} & =K_{0}\left[\frac{(1+{\bar{D}}_{out})/2}{d_{1}^{2}}-\frac{(1-{\bar{D}}_{out})/2}{d_{2}^{2}}\right],\;K_{0}=\frac{C_{0}d_{0}V_{f}^{2}}{2m} \end{array},$$

where $D_{out}$ is the one-bit output, whose value is either 1 or −1. ${\bar{D}}_{out}$ is the represented analog value, which can be got by averaging the output bit stream, whose value is continuous in [−1,1]. The expression represents the averaging effect of the bi-directional digital force, whose occurrence probability is $(1+{\bar{D}}_{out})/2$ on one side and $(1-{\bar{D}}_{out})/2$ on the other side. The corresponding parallel-plate distances of each side are $d_{1}$ and $d_{2}$. Consider there is a displacement mismatch Δd due to parasitics and a displacement change *x* due to external acceleration; then:

(12) $$\left\{\begin{array}{c} d_{1}=d_{0}+\Delta d+x\\ d_{2}=d_{0}-\Delta d-x \end{array}\right.$$

And note that, by the represented analog value ${\bar{D}}_{out}=xG_{0}\left(z\right)$, and by substituting Equation (12) in to Equation (16), then the total feedback factor β can be expressed as:

(13) $$\begin{array}{cc} \beta & =K_{0}\left[\frac{1+xG_{0}\left(z\right)}{{\left(d_{0}+\Delta d+x\right)}^{2}}-\frac{1-xG_{0}\left(z\right)}{{\left(d_{0}-\Delta d-x\right)}^{2}}\right]\\ \; & =\frac{K_{0}}{d_{0}^{2}}\left[\frac{1+xG_{0}\left(z\right)}{{\left(1+\gamma_{0}+x/d_{0}\right)}^{2}}-\frac{1-xG_{0}\left(z\right)}{{\left(1-\gamma_{0}-x/d_{0}\right)}^{2}}\right], \end{array}$$

where $\gamma_{0}=\Delta d/d_{0}$ is the mismatch factor. By using Talyor expansion, Equation (13) can be expressed as:

(14) $$\begin{array}{cc} \; & \beta =\frac{K_{0}}{d_{0}^{2}}\left(\lambda_{0}+\lambda_{1}x+\lambda_{2}x^{2}+\lambda_{3}x^{3}+\ldots \right)\\ \; & Where,\left\{\begin{array}{cc} \lambda_{0}= & \;\frac{1}{{\left(1+\gamma_{0}\right)}^{2}}-\frac{1}{{\left(1-\gamma_{0}\right)}^{2}}\\ \lambda_{1}= & -\frac{2}{d_{0}{\left(1+\gamma_{0}\right)}^{3}}-\frac{2}{d_{0}{\left(1-\gamma_{0}\right)}^{3}}+\frac{G_{0}}{{\left(1+\gamma_{0}\right)}^{2}}+\frac{G_{0}}{{\left(1-\gamma_{0}\right)}^{2}}\\ \lambda_{2}= & \;\frac{3}{d_{0}^{2}{\left(1+\gamma_{0}\right)}^{4}}-\frac{3}{d_{0}^{2}{\left(1-\gamma_{0}\right)}^{4}}+\frac{2G_{0}}{d_{0}{\left(1+\gamma_{0}\right)}^{3}}-\frac{2G_{0}}{d_{0}{\left(1-\gamma_{0}\right)}^{3}}\\ \lambda_{3}= & -\frac{4}{d_{0}^{3}{\left(1+\gamma_{0}\right)}^{5}}-\frac{4}{d_{0}^{3}{\left(1-\gamma_{0}\right)}^{5}}+\frac{3G_{0}}{d_{0}^{2}{\left(1+\gamma_{0}\right)}^{4}}+\frac{3G_{0}}{d_{0}^{2}{\left(1-\gamma_{0}\right)}^{4}} \end{array}\right. \end{array}.$$

It can be found from Equation (14) that:

1. There are odd order harmonics due to the displacement modulation effect.
2. When there is a displacement mismatch Δd, even order harmonics will come out.
3. In the expression, there are two opposite polarity terms. The total polarity is determined by the mismatch degree $\gamma_{0}$ and the loop gain $G_{0}$.

**Figure 7:**
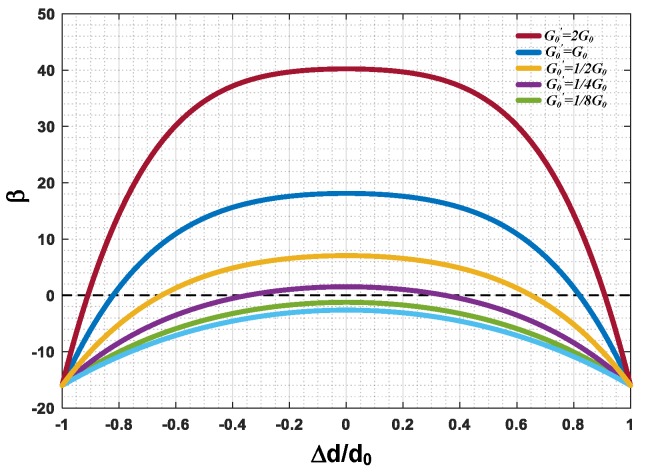
The value of β with the mismatch factor $\gamma_{0}$ at different loop gain values ($G_{0}$).

Thus, in order to avoid the undesirable change in feedback polarity, the loop gain $G_{0}$ should be above a certain limit. The lowest limit is determined by the tolerable displacement mismatch degree $\gamma_{0}$. The value of feedback factor β with the mismatch degree $\gamma_{0}$ at different loop gain $G_{0}$ is shown in Figure 7. As shown, the polarity of β will change to a negative if the mismatch factor $\gamma_{0}$ exceeds a certain range. As $G_{0}$ goes down, the tolerable range could shrink to zero, which means the system is highly unstable.

**Figure 8:**
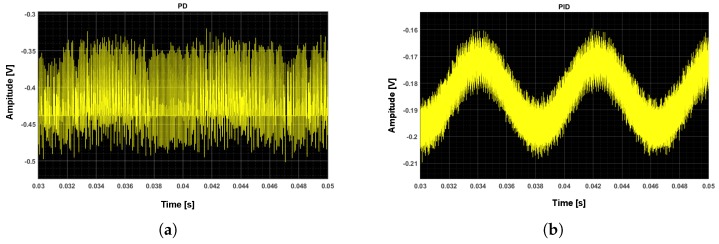
The waveform in front of electrical ΣΔ modulator: (**a**) with a traditional phase lead compensator; (**b**) with the proposed PID (proportional integral differential) compensator.

More behavior level simulation results is shown in Figure 8. A 1 g sinusoidal input acceleration is exerted onto the MEMS accelerometer, at a mismatch level of $\gamma_{0}=20\%$. The waveform in front of the electrical ΣΔ modulator is shown. As shown, the system which uses traditional phase lead compensator is unstable. However, when the compensator is changed to the proposed PID compensator, the output goes back to being stable due to the enhancement of loop gain.

### 3.3. The Digital Quantization Noise Loop

**Figure 9:**
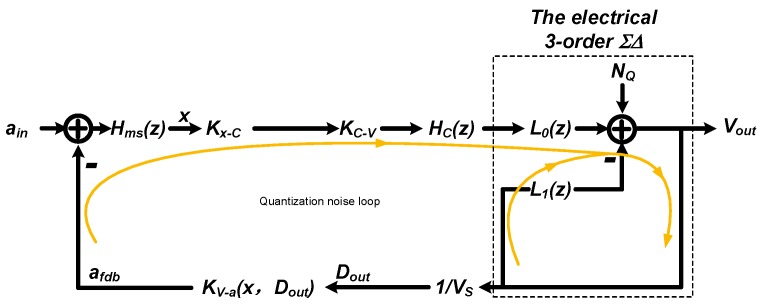
The signal flow diagram of the quantization noise loop.

The EM-ΣΔ system is also a digital output system, where the digitalization is fulfilled by the one-bit quantizer. The one-bit quantizer causes nonlinearity in two ways: (1) an indeterminate quantization gain; (2) an additional quantization noise. From the viewpoint of quantization noise, the signal flow diagram could be rearranged as Figure 9.

According the signal flow diagram shown in Figure 9, the quantization noise transfer function $NTF_{EM-\Sigma \Delta }$ of the EM-ΣΔ system can be derived as:

(15) $$NTF_{EM-\Sigma \Delta }\left(z\right)=\frac{1}{1-L_{1}\left(z\right)-L_{0}\left(z\right)L_{MS}\left(z\right)}$$

(16) $$L_{MS}\left(z\right)=K_{V-a}K_{x-C}K_{C-V}H_{ms}\left(z\right)H_{C}\left(z\right),$$

where $L_{0}\left(z\right)$ and $L_{1}\left(z\right)$ are the transfer functions of forward and feedback path of third-order Σ and Δ filters respectively; $L_{MS}\left(z\right)$ is the lumped signal transfer function of the mechanical path.

**Figure 10:**
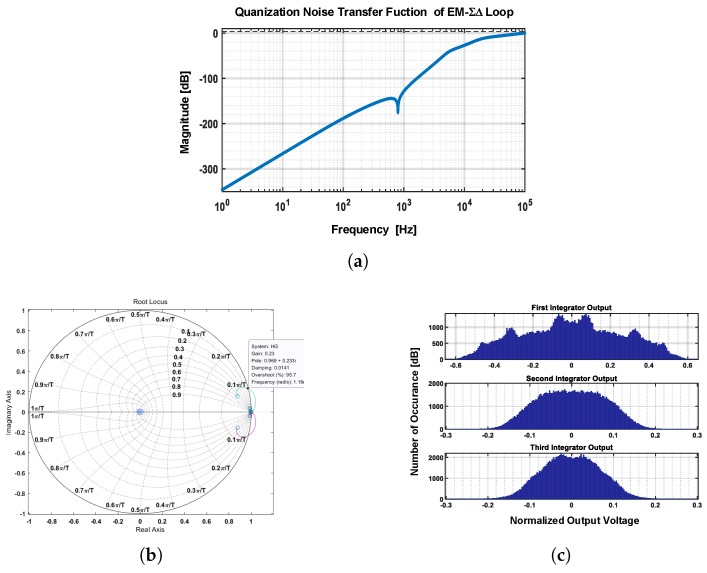
Test results of digital quantization noise loop: (**a**) noise transfer function (NTF) response; (**b**) root locus of the quantization noise loop; (**c**) the amplitude of each integrator output.

The system is further checked out by a fifth order EM-ΣΔ modulator in multiple ways, following the traditional stability criterion of purely electrical ΣΔ ADC [20,23]. The test result is shown in Figure 10. It can be found that:

1. The *Lee stability criterion*: $max|NTF_{EM-\Sigma \Delta }\left(z\right)|<1.5$ is satisfied [23] (as shown in Figure 10a).
2. The loop parameter is optimized according the root locus plot, making the stability boundary at gain = 0.23 (as shown in Figure 10b).
3. The amplitude of each critical node is observed, in case of the occurrence of overload (as shown in Figure 10c).

## 4. Implementation Details

**Figure 11:**
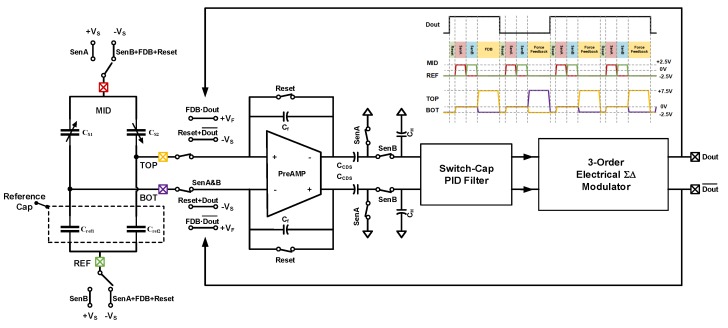
The top level implementation of the fifth-order EM-ΣΔ loop.

A fifth-order EM-ΣΔ MEMS accelerometer system was designed using proposed method. After system level synthesizing of loop parameters, circuit level implemetation is carried on. Shown in Figure 11, is the top level implementation of the fifth-order EM-ΣΔ loop. The top level switching behavior is toggling between four working phases: (1) Reset (RST); (2) positive sense (SenA); (3) negative sense (SenB); (4) force feedback (FDB). The timing diagram is also shown in Figure 11.

At first, the MEMS sensing element is broken from the interface; both the sensing capacitance bridge and the front end circuit are put into reset mode. All the charges sorted in the previous force feedback stage are clear, and each node is returned to low voltage state; thus, realizing the isolation of HV (high voltage) and LV (low voltage) isolation in time domain. After the preparing of reset phase, there are two inverted sensing phases, in which the capacitance bridge charges to invert voltages. By subtracting the sensed voltage sorted on the $C_{CDS}$, the unchanged front-end mismatch and low-frequency noise get canceled out, achieving a good low frequency noise performance. Next, the sensing element is disconnect from the front-end circuit again. A HV electrostatic voltage is exerted onto the sensing element at the logic control of output state $D_{out}$; thus, realizing the port multiplexing.

**Figure 12:**
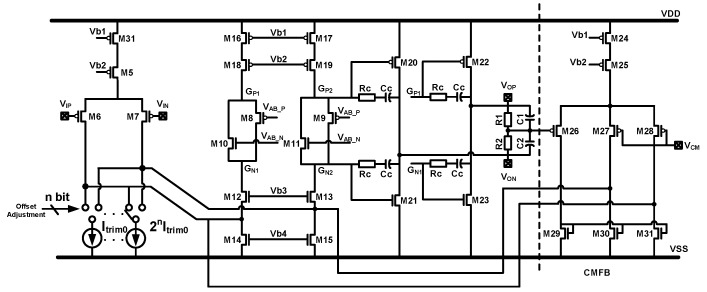
The circuit diagram of the front-end amplifier.

The circuit diagram of the front-end amplifier is shown in Figure 12. The amplifier is implemented using a fully-differential two-stage architecture. A class-AB output stage is used for driving the large front-end capacitor in sensing phase and achieving better linearity performance. Furthermore, the front-end mismatch can be tuned out by reserved trimming current source $I_{trim}$ array. The trimming array is binary coded, and can be accessed by preserved serial peripheral interface (SPI) interface.

**Figure 13:**
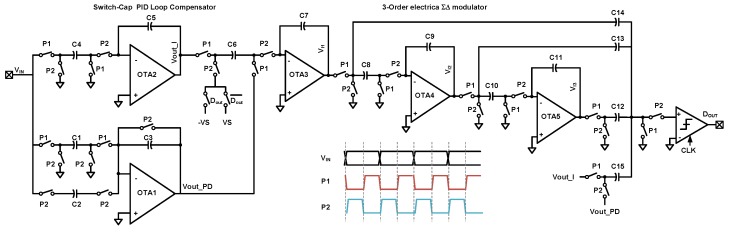
The signal flow diagram of the quantization noise loop.

The circuit diagram of back-end discrete-time PID compensator and third-order electrical ΣΔ modulator is shown in Figure 13. For simplicity, the diagram is shown in single-ended version, and fully-differential version could easily be derived. The third-order electrical is implemented using existing feed-forward architecture; the switching logic and timing diagram are also shown in Figure 13.

The proposed discrete-time PID compensator is implemented by OTA${}_{1,2}$ and capacitors C${}_{1∼5}$. The transfer function of it can be derived by the flowing charge balancing equation:

(17) $$\left\{\begin{array}{c} V_{IN}\left[n-1\right]C_{4}+V_{out\_I}\left[n-1\right]C_{5}=V_{out\_I}\left[n\right]C_{5}\\ V_{IN}\left[n-1\right]C_{1}=V_{IN}\left[n\right]C_{2}+V_{out\_PD}\left[n\right]C_{3} \end{array}\right.,$$

(18) $$\left\{\begin{array}{c} V_{out\_I}=\frac{C_{4}}{C_{5}}\frac{z^{-1}}{1-z^{-1}}V_{IN}\\ V_{out\_PD}=-\left[\frac{C_{1}-C_{2}}{C_{3}}+\frac{C_{1}}{C_{3}}\left(1-z^{-1}\right)\right]V_{IN} \end{array}\right.,$$

where the $V_{out\_I}$ and $V_{out\_PD}$ are the output voltages of integral and proportional-derivative stages respectively. The summation of them is realized on the sampling capacitance $C_{6}$ of the first ΣΔ integrator. In the sampling phase, the charge sampled onto $C_{6}$ can be expressed.

(19) $$\begin{array}{cc} Q_{C_{6}} & =\left(V_{out\_I}-V_{out\_PD}\right)C_{6}\\ & =\left[K_{P}+K_{I}\left(\frac{z^{-1}}{1-z^{-1}}\right)+K_{D}\left(1-z^{-1}\right)\right]V_{IN}C_{6} \end{array}$$

Then the PID coefficients $K_{P}$, $K_{I}$, $K_{D}$ can be given in Equation (20), and can be adjusted by the capacitor ratio between $C_{1∼5}$.

(20) $$\left\{\begin{array}{c} K_{P}=\frac{C_{1}-C_{2}}{C_{3}}\\ K_{I}=\frac{C_{4}}{C_{5}}\\ K_{D}=\frac{C_{1}}{C_{3}} \end{array}\right.,$$

## 5. Results and Discussion

**Figure 14:**
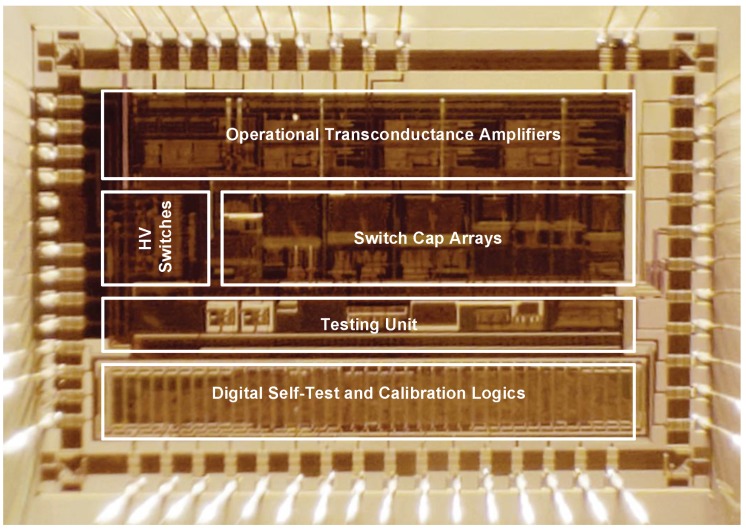
The micro-photograph of the die of the ASIC.

The proposed EM-ΣΔ readout interface for MEMS accelerometers was designed and fabricated in 0.35 μm CMOS-BCD process with a chip area 4.0 × 3.8 mm. The micro-photograph of the die of the application specific integrated circuit ASIC is shown in Figure 14. The interface ASIC contains HV (high-voltage) switches, fully-differential operational transconductance amplifiers, switch-capacitor arrays, a testing interface, a digital timing sequence and on-chip BIST (built in self-test) and calibrating logic. The clock frequency is 500 kHz, which is referenced from an off-chip quartz crystal. The ASIC consumes 35 mW from low noise ±2.5 V DC supply. The HV driving voltage is powered from an off-chip 12 V voltage source.

**Figure 15:**
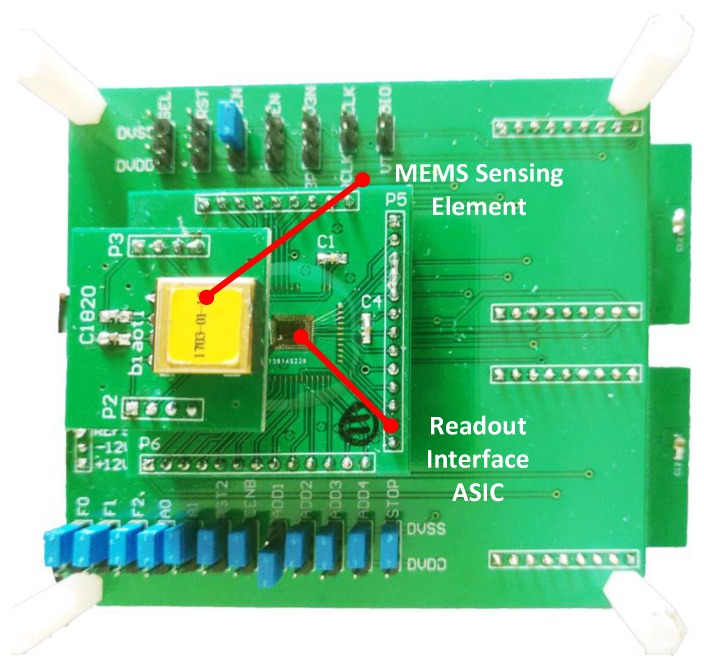
The photograph of the testing mother board with a ASIC and MEMS accelerometer mounted.

The interface ASIC was tested with a vacuum packaged MEMS accelerometer. The MEMS accelerometer has a resonating frequency $f_{0}$ at 1 kHz and a quality factor *Q* as high as 200. The interface ASIC and MEMS accelerometer are mounted to a PCB mother board (as shown in Figure 15) for coordinate testing. The key nodes inside the interface ASIC are led outside for testing by a high speed buffer, in the case of disturbing the loop characteristic. The critical loop parameters (e.g., $K_{P},K_{I},K_{D}$ and input offset) can be tuned by on-chip capacitance arrays. The test point selection and parameter adjustment can be fulfilled online by a digital SPI interface between testing mother board and readout ASIC.

**Figure 16:**
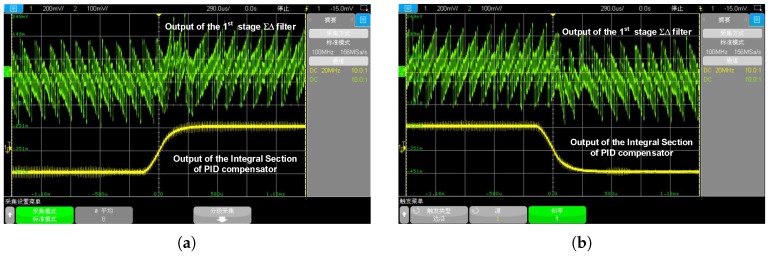
The step response of the EM-ΣΔ system: (**a**) the step response of rising edge; (**b**) the step response of the falling edge.

Shown in Figure 16, is the step-response of the EM-ΣΔ loop. This functional test is full-filled by BIST function. The on-chip BIST excitation source generates a square-wave voltage excitation; the amplitude is 5 V${}_{pp}$, providing an equivalent acceleration of approximate 5 g (calculated by electrostatic force Equation (2)). The waveform shows the rising edge and falling edge response of the integral section of PID compensator and the first stage of the electrical ΣΔ filter. It can be found that, the proposed PID loop compensator provides sufficient electrical damping to the highly under-damped MEMS sensing element. Thus results in an over-damped step response. The proposed system shows excellent stability performance.

**Figure 17:**
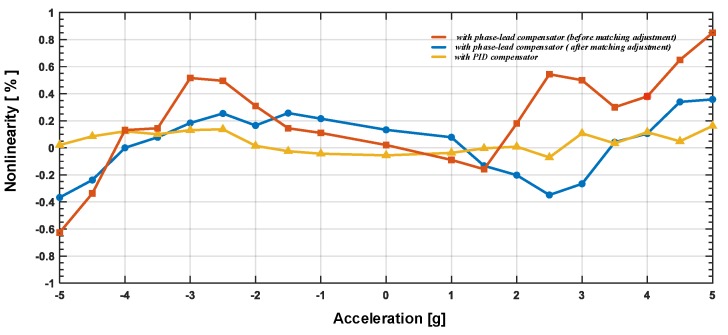
The nonlinearity test result of the EM-ΣΔ accelerometer.

The full-scale nonlinearity was tested by a series of DC tests; the nonlinearity test result is shown in Figure 17. The input DC accelerations are generated by electrostatic force. As shown, the initial linearity performance is over 0.8%. This is due to the fact that the parasitic capacitance mismatch level is high, which deflects the proof mass from its central balanced position. This deflection will cause asymmetry in digital feedback force (as shown in Equation (14)) and induce nonlinearity in the output transfer function. The mismatch can be compensated by tuning the preserved compensating capacitors and the trimming current source in the front-end amplifier. After matching adjustment, the nonlinearity could be effectively reduced. Compared by traditional phase-lead compensator, the proposed PID compensator achieves a better linearity performance, due to the improvement in in-band gain. As shown, the nonlinearity of proposed architecture achieves a nonlinearity level below 0.2%, which is about 1/3 of traditional phase-lead compensator. And the tendency of increased nonlinearity with higher input acceleration is not obvious; this means the residue displacement induced error is effectively suppressed.

**Figure 18:**
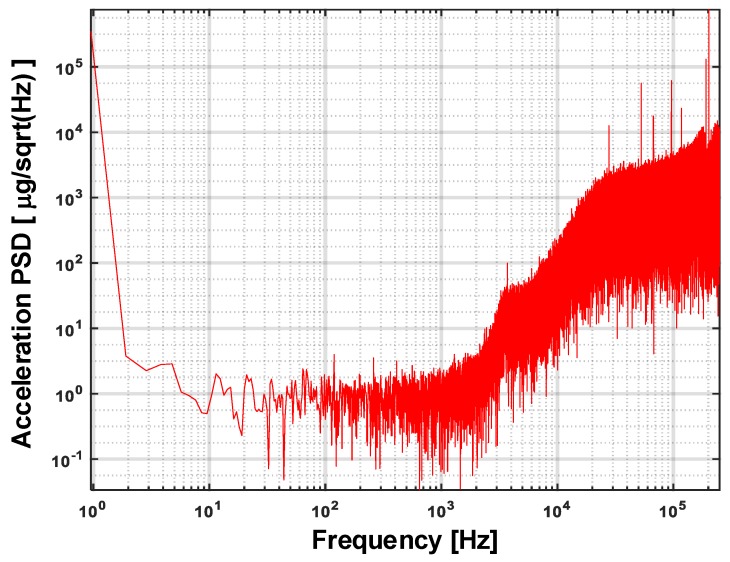
The input referred noise spectrum density of the proposed EM-ΣΔ accelerometer.

The output bit stream of the EM-ΣΔ accelerometer is captured by logic analyzer Agilent 16804A. Using the captured bit stream, the input referred noise spectrum density of the system in a static 1 g environment is calculated by 524,288 point FFT (fast Fourier transform). The result is shown in Figure 18, with magnitude normalized to $\mu g/\sqrt{\mathrm{Hz}}$. It was found that the proposed system achieved a noise floor as low as $1\;\mu g/\sqrt{\mathrm{Hz}}$ in a frequency range extending up to 1 kHz. With a full scale range of ±5g, a dynamic range of 140 dB was achieved.

## 6. Conclusions

This paper provides a straightforward viewpoint from which to analyze the EM-ΣΔ loop. The complicate electromechanical loop was analyzed and discussed in three aspects: the signal loop; the local positive feed-forward path; the quantization noise loop. We pointed out that the synthesizing procedure should be taken step by step.

At each design stage, by taking separate consideration and proper simplification, the loop synthesizing problem is made clear and straightforward. By the proposed design step, the designer could avoid facing a mixture of the knotty problem at first and get a quick intuitive understanding of the loop behavior at once.

A fifth-order EM-ΣΔ accelerometer was realized using proposed design methodology. By a clear understanding the signal loop and local positive feed-forward path, a more effective discrete-time PID loop compensator is used to instead traditional phase-lead one. The test results shows that the proposed PID compensator provides sufficient electrical damping to the highly under-damped MEMS accelerometer. The linearity is improved compared to traditional phase-lead system. A noise floor of 1 $\mu g/\sqrt{\mathrm{Hz}}$ was achieved, with a bandwidth 1 kHz and a dynamic range of 140 dB.
